# Comparative Health Behaviour of Young People with Disabilities in Hungary: A Cross-Sectional Study

**DOI:** 10.3390/children11050589

**Published:** 2024-05-13

**Authors:** Agota Barabas, Attila C. Nagy, Viktoria Pazmany, Anita K. Grestyak Molnarne, Agnes Nemeth, György Jona, Agnes Santha, Peter Takacs, Emil Toldy-Schedel, Renata Javorne Erdei

**Affiliations:** 1 Department of Health Methodology and Prevention, Faculty of Health Sciences, University of Debrecen, 4400 Nyíregyháza, Hungary; pazmany.viktoria@etk.unideb.hu (V.P.); grestyak.anita@etk.unideb.hu (A.K.G.M.); erdei.renata@etk.unideb.hu (R.J.E.); 2 Department of Health Informatics, Faculty of Health Sciences, University of Debrecen, 4032 Debrecen, Hungary; attilanagy@med.unideb.hu; 3 Institute of Psychology, Eötvös Loránd University, 1053 Budapest, Hungary; nemeth.agnes@ppk.elte.hu; 4 Department of Social Sciences, Faculty of Health, University of Debrecen, 4400 Nyíregyháza, Hungary; jona.gyorgy@etk.unideb.hu; 5 Department of Applied Social Sciences, Faculty of Technical and Human Sciences, Sapientia Hungarian University of Transylvania, 540485 Targu Mures, Romania; santhaagnes@ms.sapientia.ro; 6 Department of Health Informatics, Faculty of Health Sciences, University of Debrecen, 4400 Nyíregyháza, Hungary; takacs.peter@etk.unideb.hu; 7 St. Francis Hospital of Budapest, 1021 Budapest, Hungary; titkarsag@szentferenckorhaz.hu

**Keywords:** health, health behavior, disability, Visegrad group

## Abstract

The health status of Hungary’s population is unfavorable, with significant differences in health indicators not only compared to the EU15 but also to the Visegrad countries. Unfavorable health indicators can be disproportionate and particularly affect vulnerable groups, such as people with disabilities. In this study, we set out to compare the health behavior of disabled youth and youth with typical development in Hungary. We also aimed to compare the health behavior of adolescents in the Visegrad countries. The eating habits of both groups of young people we examined are unfavorable. Adolescents with disabilities experience a significantly higher rate of school stress than children with typical development in Hungary. The prevalence of somatic complaints and parameters of poor mental well-being are significantly higher in Hungary than in the other Visegrad countries. The results indicate that additional interventions are needed in Hungary and that differentiated, professional health promotion is needed for young people with disabilities. The researchers recommend extending the study to disabled adolescents living in Visegrad countries, on the basis of which an injury-specific health promotion methodology could be developed with international interprofessional cooperation.

## 1. Introduction

The health status of the Hungarian population is among the worst in the European Union (EU). Although there has been an improvement in health indicators over the last two decades, there are significant differences not only in relation to the EU15 but also to the Visegrad countries (Czech Republic, Poland, Slovakia, hereafter V3) [1,2]. The Visegrad countries include four Central European countries, the Czech Republic, Hungary, Poland, and Slovakia [3]. Although life expectancy at birth has been on a steadily increasing trend, it is still lower than in the V3 countries: in 2021, life expectancy in Hungary is expected to be 77.8 years, in the Czech Republic it is 80.5, in Poland it is 79.6, and in Slovakia it is 78.2 years, compared to 80.1 years in the EU27 [4]. The number of preventable deaths that could have been avoided with primary prevention interventions in Hungary is the highest in the EU. In 2020, 350 deaths per 100,000 people could have been avoided, compared to 275 in Poland, 218 in the Czech Republic, and 262 in Slovakia [1,5,6,7]. In Hungary, ischemic heart disease and brain diseases have been the leading causes of death for many years, followed by cancer deaths, which is similar in the V3 countries [8]. In terms of early mortality, i.e., under 65 years of age, cancer is the main cause of death in Hungary, which had the highest malignant cancer mortality rate in the EU in 2019 [9]. The country also has a poor demographic profile, which is linked to the health status of the population. Hungary’s population is steadily declining, reflecting an aging society [2].

In 2019, nutritional risk factors were responsible for around half of all deaths in Hungary, higher than the EU average of 17%. In Hungary, tobacco accounted for 21% of all deaths; in Poland, it was responsible for about 20%. Obesity is a problem in all Visegrad countries. In 2019, among EU member states, 19% of adults in the Czech Republic and 16% of the adult population in Slovakia were obese, similar to the EU average. The obesity rate in Poland’s adult population lies above the EU average with 18.5%, whereas in Hungary it is extremely high (33%) [1,5,6,7]. High overweight/obesity rates, poor dietary habits, and risky behaviors are already present in childhood. The Health Behaviour in School-aged Children (HBSC) survey is an internationally collaborative youth survey that has been in place for more than 36 years [10]. The international results for 2018 show that physical activity levels are poor in most countries, with less than one in five adolescents completing the global physical activity target of at least 60 min of physical activity per day (MVPA recommendation) [11]. Walking, cycling, and active play, which used to be part of every day, have disappeared from the daily lives of adolescents, but the amount of time spent in sedentary states has increased [12]. One in five adolescents is overweight or obese, with higher rates in younger age groups and among boys. Children from families with a better socio-economic background reported better health and well-being, higher physical activity levels, and more favorable dietary habits [11].

In the present study, we aimed to compare the health behaviors of adolescents with disabilities and adolescents with typical development in Hungary. Furthermore, we aimed to compare adolescent health behavior in the Visegrad countries. 

Launched by the research team in 2021, the aim of the research was to explore the health behavior of young people aged 12–18 with disabilities in the Northern Great Plain Region of Hungary and the factors influencing it. Many diseases are established during childhood and even earlier, in fetal life; therefore, it is necessary to examine the situation of mothers and their access to health care from conception onwards, as a good start in life is a prerequisite for good health in childhood [13]. 

Poor health indicators can have a disproportionate impact and particularly affect vulnerable groups, which include people with disabilities whose health indicators are worse than the average.

More than 1 billion people live with a disability, affecting 15% of the world’s population. People with disabilities have greater health care needs, health problems and more difficulties in accessing health care. People with disabilities are more likely to have an inadequate nutritional status, three times more likely to suffer from diabetes mellitus, and three times more likely to be denied health care [14]. The proportion of women with disabilities in EU Member States was higher than that of men in 2022 [15]. According to the Hungarian Central Statistical Office (KSH), 408,021 people in Hungary had some type of disability in 2016, representing 4.3% of the population [16]. In Hungary, in the North Great Plain Region, 2042 persons aged 12–18 years live with a disability, 984 persons in Szabolcs-Szatmár-Bereg county, 444 persons in Jász-Nagykun-Szolnok county, and 614 persons in Hajdú-Bihar county [16].

Children and adolescents with disabilities often have poorer health outcomes than their typically developing peers [17]. They are more likely to be affected by obesity and diabetes, have a higher incidence of developmental disorders, heart and respiratory diseases, mental health problems, and premature mortality [18]. Children and young people with disabilities are at higher risk of developing cardiovascular disease than their typically developing peers [19]. Adults with IDs are less physically active than the general population [20]. 

Differences in health status are known to be associated with the disability itself, e.g., congenital heart defects and premature dementia in children with Down’s syndrome (DS). Children and adolescents with DS have higher overweight/obesity rates than their typically developing peers. The likely causes of obesity in overweight young people with DS may include increased leptin levels, lower prealbumin levels, lower resting energy expenditure, poor dietary preferences, and comorbidities. However, in young people with DS, the presence of obesity increases the risk of dyslipidemia, obstructive sleep apnea, hyperinsulinemia, and gait disturbances [17]. In this research, adolescents living with ASD are included in the broader category of intellectual disability. Children with autism spectrum disorder (ASD) may have a higher incidence of gastrointestinal disorders. There is a higher prevalence of eating problems, which occur in 46–89% of children with ASD, and a higher rate of obesity [21,22]. In general, in the case of all intellectual disabilities, somatic problems are often complex and occur at a higher rate than in the general population. Intellectual disability is often associated with multimorbidity, and children with intellectual disabilities (IDs) have a shorter life expectancy. Thus, the co-existence of abnormal psychiatric and somatic conditions is common. The interaction of existing disability and comorbidities makes the health status of people with intellectual disability even more vulnerable [16,22,23]. There are, however, differences that are not explained by the biological basis of health status but are associated with a risk of poorer health. Such social determinants of health include poverty and social exclusion. Access to health promotion, education, and some health services is difficult for young people with disabilities, and the stigma associated with different developmental trajectories is a frequent experience for them. Children with disabilities are often characterized by multiple disadvantages. In addition to existing disability, health status is also affected by the income status of the country or family and by ethnicity, which can reduce but also exacerbate disadvantage and social exclusion [16]. Financial status also affects a child’s physical activity, with children from more affluent families having a higher rate of regular sport participation. Parents’ educational attainment has an impact on children’s diet [24]. Health literacy, health awareness, and financial status vary among children’s families, which places a strong emphasis on school health and school–parent partnerships to promote healthy lifestyles [25].

## 2. Materials and Methods

### 2.1. Aim of the Research

**Figure 1:**
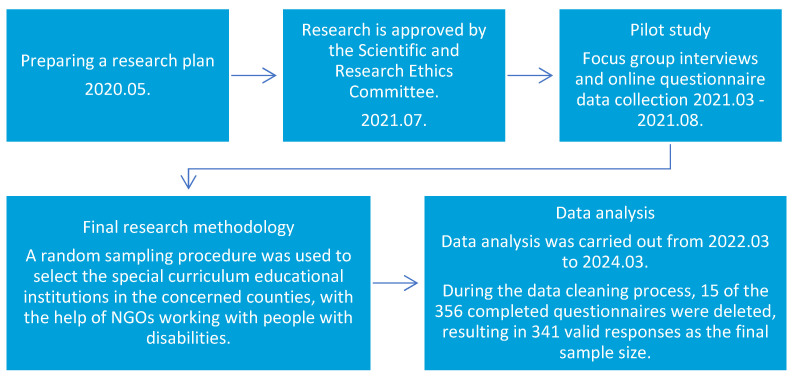
Flowchart of the research process (own editing).

The study has two objectives: firstly, to examine the health behavior of young people with disabilities aged 12–18 living in the Northern Great Plain region. The aim is to compare the health behavior and health indicators of adolescents with disabilities with those of the general adolescent population in Hungary. The second research objective is to compare data from the HBSC survey in the Visegrad countries. The survey was conducted in preparation for a needs-based intervention. In our study, special schools in the counties covered by the research were randomly selected through a cluster sampling procedure. Everyone from a given school who met the selection criteria (age 12–18, disability, living in the Northern Great Plain Region) and who gave prior consent to the study was included in the sample. Children and parents were both asked for consent. Those who did not live in the region, did not suffer from a disability, and were not in the specified age group were excluded from the sample. The gross sample consisted of pupils from 12 special schools and from NGOs working with people with disabilities. Of the schools selected, 366 agreed to participate in the survey, giving a response rate of 93.81%. Finally, 356 paper questionnaires were collected. During the computer data entry and cleaning process, a further 15 questionnaires were deleted from the sample (e.g., 50% of the questions were not answered, due to apparently frivolous responses). The final, implemented sample size consists of 341 questionnaires (Figure 1). In the vast majority of cases, the presence of an interviewer was required during data collection. The age group included in our research was 12–18 years. From the HBSC research sample, we selected the results of young people in the 13, 15, and 17 age groups. From the total disabled adolescent population interviewed by us, those 109 respondents that matched the age groups also targeted by the HBSC research were selected to be included in this study. Our results were also compared with the HBSC 2018 data for Hungary, as well as the HBSC survey data for the Visegrad countries.

Prior to the design of the final survey, a pilot focus group study was conducted with 23 young people with disabilities aged 12–18 years on a total of three occasions in April and May 2021. Subsequently, an online questionnaire data collection was conducted between 1 July and 31 August 2021, during which a total of 176 questionnaires were completed. A pilot study was conducted to improve the questionnaire.

In our final survey, special schools in the counties covered by the research were selected randomly using a cluster sampling procedure. 

In the counties concerned, 20 special curriculum schools were selected using a random sampling procedure. The heads of the institutions were contacted for permission to conduct the research. Finally, 12 schools agreed to participate in the research. As several schools declined to participate in the research, in order to increase the sample size, we sought the help of NGOs working with people with disabilities to reach the target group. 

In examining the associations of the different characteristics with age and gender, statistical tests justified the clustering of response categories. The response options for questions about food and drink consumption were “never”, “less than weekly”, “weekly”, “2–4 times a week”, “5–6 times a week”, “once a day”, “several times a day”, “less than weekly”, “at least weekly but not every day”, and “at least once a day”. Response categories to questions on physical activity were combined as follows: responses of “none” and “about half an hour” were combined into a category of less than 4 h, and responses of “about 4–6 h” and “7 h or more” were combined into a category of “at least 4 h”. For the question “physical activity in the last 7 days”, “0 days” and “1 day” have been merged, “2 days”, “3 days”, and “4 days” have been merged, and the answer options “6 days” and “7 days” have been added to the third category. The categories for breakfast on school days were changed as follows: “never” and “once a week” were merged, “twice” and “three times” were merged into the second category, and “four times” and “every day” were moved to a third category.

### 2.2. Method of Data Collection

During the investigation, we combined theoretical research with empirical questionnaire research in an interdisciplinary approach. In our self-developed questionnaire, we adapted certain blocks of questions from the validated questionnaire of the HBSC research to assess eating habits, dental care, and physical activity. Data collection covered the following topics. Demographic data: age, gender, parents’ education, place of residence, and self-assessed financial situation. Questions about health behavior: physical activity, eating habits and dental care, smoking habits, alcohol consumption, leisure time, age, gender, education, and place of residence. 

### 2.3. The Ethical Background of the Research

The questionnaire was completed anonymously; the persons participating in the research cannot be identified. The research was carried out in compliance with the applicable law, professional guidelines, and recommended ethical codes. The rules for querying and collecting the questionnaire, processing, storage, and database management complied with the relevant legislation. The research was approved by the Scientific and Research Ethics Committee, license number: IV/5706-1/2021/EKU.

### 2.4. Statistical Analysis 

In the first phase of the analysis, the characteristics of the sample were calculated (descriptive statistics, frequencies, proportion in the sample). During the more complex tests, the subgroups were compared with the two-sample *t*-test, ANOVA, and Mann–Whitney tests and the relationships between the variances were estimated by calculating correlation coefficients. Two ratio Z-tests were used to compare proportions. A *p*-value < 0.05 was considered significant. Data were analyzed using Intercooled Microsoft Excel 2007, SPSS V24, and Stata v17 [26].

### 2.5. Presentation of the Sample

The sample sizes for the Visegrad countries from the HBSC 2018 database were as follows: Czech 11,470, Hungary 3739, Poland 5181, and Slovakia 4621 people. The Hungarian disabled children (hereafter HUDC) sample was 109 people. The total sample was 25,120. In our survey, in the sample of young people with disabilities, boys were over-represented (57.8% vs. 42.2%). The highest proportion of young people in the sample was aged 15 years. In terms of disability type, participants were most affected by mild intellectual disability, followed by those with mild autism spectrum disorder and those with mild learning disability. The lowest proportion was young people with hearing impairment. A total of 66.1% of pupils live in a city, and 80.7% live with their families. A total of 9.2% of respondents live in dormitories, 2.8% in children’s homes, and 7.3% in foster care (Table 1).

## 3. Results

### 3.1. Life Satisfaction, Physical Activity, and Dietary Habits of Adolescents Living with Disabilities in Hungary

We asked respondents to rate their satisfaction with their life on a Likert scale of 0 to 10, where 0 was the worst possible life and 10 was the best possible life. A total of 21.1% of HUDC rated their life as the best possible, whereas 0.9% as worst possible. A total of 15.6% of young people rated their life as 5, and a minority were less satisfied (Table 2).

During the 7 days prior to completing the questionnaire, the highest proportions of HUDC had not engaged in physical activity at all (22.9%) or on 1 day only (21.1%). A total of 10.1% of respondents were physically active every day (Table 3).

A third of HUDC had not performed any vigorous physical activity in the last seven days. At least half an hour of daily physical activity was performed by 21.1% of the respondents (Table 4).

Slightly over half of HUDC (54.1%) eat breakfast on all 5 school days, whereas 14.7% of respondents never eat breakfast (Table 5).

A total of 2.8% of HUDC never eat fruit and 9.2% never eat vegetables in general. A total of 16.5% of respondents eat fruit and vegetables daily. The proportion of people who consume fruits and vegetables more than once a day is lower. A total of 22.9% of respondents eat sweets every day. Cola or other soft drinks are consumed daily by 12.8% and more than once a day by 10.1% of HUDC (Table 6).

### 3.2. Life Satisfaction, Physical Activity, and Dietary Habits of Young People with Disabilities in Hungary in Relation to Age and Gender

**Figure 2:**
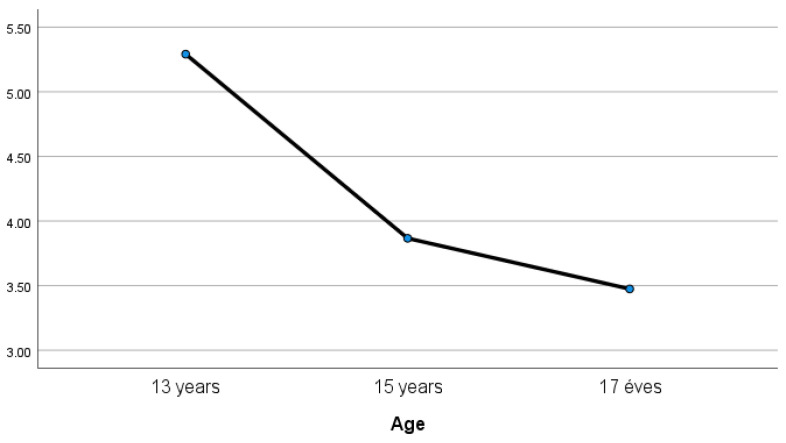
Satisfaction with life mean N = 109 (own editing).

Life satisfaction decreased significantly with age (ANOVA, *p* = 0.018; Figure 2). On average, life satisfaction was 5.3 at age 13 and as low as 3.5 at age 17. Comparing genders, no significant association with life satisfaction was found (two-sample *t*-test, *p* = 0.762). On average, boys and girls had 3.9 and 4.1 levels of life satisfaction, respectively.

No significant association was found between weekly physical activity and age. There was no significant association between gender and physical activity, or between gender and vigorous physical activity.

No significant association was found between gender and the frequency of having breakfast on school days. During a period of 5 days, a total of 15.9% of boys and 30.4% of girls never or very rarely eat breakfast. A total of 58.7% of boys and 56.5% of girls have breakfast 4–5 times a week. Age was not an influential factor in the frequency of breakfast consumption.

No significant association was found between gender and fruit consumption either. Vegetable consumption also showed no association with gender. Eating sweets was not significantly associated with gender. Similarly, there was no significant association between gender and Cola or other soft drinks consumption.

There is no significant association between age and fruit consumption, or between age and vegetables consumption. Age and the consumption of sweets are also not related to each other. Age and the consumption of Cola and other soft drinks are not related to each other either.

### 3.3. Comparison of Health Behavior of Young People with Typical Development in Hungary and Young People with Disabilities in Hungary

#### 3.3.1. Eating Behaviors and Oral Health

In Hungary, a higher proportion of HUDC ate breakfast on school days on all 5 days (45.39% vs. 54.13%). The regular consumption of fruit and vegetables (several times a day) was also lower among HUDC than among adolescents with typical development. The consumption of sweets several times a day was higher among children with typical development (11.08%) than among HUDC (7.34%). A total of 24.44% of the average child population and 22.94% of the HUDC drank Coca-Cola or other soft drinks every day. Brushing teeth several times a day is more common among young people with typical development (61.12%), with only 28.44% of HUDC brushing their teeth several times a day (Table 7).

#### 3.3.2. Physical Activity, Body Image, Health Status

Prior to completing the questionnaire, a higher proportion of young people with typical development were physically active than HUDC, and they were also more active 4–6 times a week than HUDC. However, only 6.42% of HUDC are overweight or obese compared to a higher proportion of the other group (25.74%). The proportion of children who are too thin is 6.42% for HUDC compared to 12.29% for typically developing children in Hungary. A total of 32.63% of typically developing children and 22.94% of HUDC feel too fat. A total of 6.42% of HUDC and 12.29% of typically developing children consider themselves too thin. A total of 32.63% of youth with typical development and 22.94% of HUDC feel too fat (Table 8).

#### 3.3.3. Family Context

HUDC have lower rates of mother and father living with their child than typically developing children in Hungary, but the difference is not significant for either parent. A lower proportion of mothers of HUDC have a job (57.80%) than mothers of typically developing youth (86.28%). The proportion of fathers with a job is significantly higher among typically developing youth (92.30% vs. 73.39%) (Table 9).

#### 3.3.4. Life Satisfaction, School Experience, Mental Well-Being

There is no significant difference in life satisfaction between the Hungarian general population and HUDC, with a slightly higher proportion of HUDC rating their lives as the best possible (21.10% vs. 17.96%). Typically developing young people were significantly more likely (*p* < 0.011) to feel depressed at least once a month (71.76%) than HUDC (60.55%). A total of 61.80% of Hungarian children had a tummy ache at least once a month, which is significantly higher than in HUDC with a rate of 44.95%. No significant difference was found for back pain. In terms of headache, a significantly higher proportion of adolescents (65.72%) complained of headache compared to HUDC (43.12%). The rate of dizziness complaints is also higher among children with typical development than among HUDC, but the difference is not significant. Sleep difficulties affected both groups to an approximately similar extent. However, schoolwork is significantly more stressful for HUDC (56.88%) than for typically developing young people in Hungary (7.20%) (Table 10).

#### 3.3.5. Drunkenness

A higher proportion of HUDC had been drunk 2–3 times in their lifetime than typically developing children in Hungary, although the difference is not significant (Table 11).

### 3.4. Comparison of Health Behavior in the Visegrad Countries

#### 3.4.1. Eating Behaviors and Oral Health

Among the countries included in the analysis, the proportion of young people who regularly ate breakfast on weekdays was highest in Poland (61.24%), which is significantly (*p* < 0.001) higher than in Hungary (45.39%), the Czech Republic (52.88%), and Slovakia (45.99%). The proportion of young people who ate fruit and vegetables several times a day is low in all countries. In Slovakia, a significantly higher proportion (*p* < 0.001) of young people consume vegetables more than once a day than in Hungary (18.72% vs. 13.55%). The consumption of Cola and sugary drinks is significantly higher (*p* < 0.001) in Hungary (24.44%) than in the V3 countries (13.76% of young people in the Czech Republic, 16.27% in Poland, and 21.25% in Slovakia consumed such drinks at least once a day). Brushing teeth more than once a day was significantly higher in the Czech Republic (*p* < 0.001) compared to the other Visegrad countries (Table 12).

#### 3.4.2. Physical Activity, Body Image, Health Status

A significantly higher proportion (*p* < 0.001) of adolescents in Slovakia (23.05%) than in the Czech Republic (18.29%) and Poland (17.16%) were physically active daily. Also, a significantly higher proportion of adolescents in Slovakia than in Hungary (*p*: 0.016) engaged in physical activity on all 7 days before completing the questionnaire (*p*: 0.016). The highest proportion of adolescents (51.27%) engaged in vigorous physical activity at least 4–6 times per week was also found in Slovakia (*p* < 0.001), which is significantly higher than in the Visegrad countries (*p* < 0.001). Overweight/obesity was significantly higher (*p* < 0.001) in the average Hungarian population compared to the other Visegrad countries. Among minority Hungarians residing in Romania (Transylvania), Slovakia (Southern Slovakia), Serbia (Vojvodina) and Ukraine (Transcarpathia), the proportion of those who have never engaged in exercise varies between 34% (in Serbia) and 53% (in Romania), with an additional one-fifth of the population doing exercise less than once in a week. In each region, regular physical activity tends to be more prevalent among those with higher education and decreases with age [27]. No significant difference is found for thinness. More than 12% of young people found themselves too thin. The highest proportion of young people considering themselves as fat was found in Poland (39.09%), which is significantly higher (*p* < 0.001) compared to Hungary (32.63%), the Czech Republic (25.36%), and Slovakia (24.43%). The proportion of students who consider their health to be excellent is the highest in Slovakia (27.69%) (Table 13).

#### 3.4.3. Life Satisfaction, School Experience, Mental Well-Being

Overall, more than 14% of young people were satisfied with their lives and considered it the best life possible. A total of 71.76% of Hungarian children in the HBSC survey felt depressed at least once a month, which was higher (*p* < 0.001) compared to other Visegrad countries. Also, Hungarian children complained of tummy aches at least once a month in the highest proportion (61.80%), which is significantly higher (*p* < 0.001) than in the Czech Republic, Poland, and Slovakia. The prevalence of back pain is also highest in Hungary (45.49%), but its occurrence is above 35% in the other Visegrad countries, too. Among the complaints, the rate of monthly headache was significantly (*p* < 0.001) higher in Hungary compared to the other countries studied. The prevalence of dizziness complaints was lowest among Czech adolescents (16.91%), with no significant differences, but highest among Hungarians (33.30%). A total of 56.91% of Hungarian adolescents had difficulty falling asleep at least once a month, which is significantly higher (*p* < 0.001) than in the case of young people in Poland and Slovakia. Polish young people are most likely to find schoolwork very overwhelming (14.44%) (Table 14).

#### 3.4.4. Drunkenness

A significantly higher proportion (*p* < 0.001) of young people living in Hungary had been drunk at least 2–3 times than Polish and Slovakian young people (Table 15).

#### 3.4.5. Family Context

The share of pupils living with their mother is highest among the Polish young people (96.28%), which is significantly higher than in the other Visegrad countries. The rate of young people living with the father is lower than those living with the mother in all countries, but is significantly higher in Poland. The proportion of mothers having a paid job is significantly highest in Poland (79.63%). The proportion of fathers with a job is highest in Slovakia (93.51%) (Table 16).

## 4. Discussion

The highest proportion of HUDC considered their health to be at its best, which declined significantly with age. Nearly half of the children had 0 or 1 day of physical activity in the 7 days prior to completing the questionnaire. On the positive side, the majority ate breakfast on every school day. However, the consumption of fruit and vegetables was low, with less than 10% eating these foods more than once a day. Eating habits and physical activity were not influenced by age and gender.

This study had two objectives. One was to compare the health behavior of HUDC with data from the 2018 HBSC of Hungarian young people with typical development. The findings showed that the eating habits of both populations were unfavorable, with fruit and vegetable consumption and dental care being more unfavorable among HUDC, but the difference was not significant. In terms of somatic complaints, depression, stomachaches, and headaches were more prevalent among typically developing children. HUDC are slightly less physically active, but the ratio is also unfavorable for the average Hungarian child population. Among the risk behaviors examined, drunkenness was slightly higher among HUDC. Schoolwork, on the other hand, depressed HUDC at a significantly higher rate. 

When designing intervention programs, it should be taken into account that stress from schoolwork and milk production is positively and significantly related to symptoms of depression and inversely related to life satisfaction [28].

Because of the high prevalence of somatic problems, recognition and treatment are essential to prevent psychological problems [22].

HUDC had a lower proportion of mothers living with their families and the same proportion of fathers living with their families. HUDC had a lower proportion of mothers with a paid job. Among typically developing Hungarian youth, the proportion of fathers with a job is significantly higher for HUDC.

Another aim of this study was to compare data from the HBSC survey in the Visegrad countries. It is clear that eating habits were poor in all Visegrad countries. The consumption of fruit and vegetables several times a day was lowest in Hungary, but also low in the other Visegrad countries. The consumption of vegetables several times a day was also lowest in Hungary. The consumption of Cola and soft drinks was significantly higher, and brushing teeth several times a day was the lowest in Hungary. During the 7 days preceding the survey, Hungary had the lowest physical activity rate. Adolescents living in Slovakia had the highest rate of physical activity during the survey period. Hungary had the highest proportion of obese young people. In contrast, when looking at self-assessed body image, Polish adolescents had the highest rate of self-assessed obesity compared to other countries by a significant margin. Symptoms related to physical and mental well-being, such as dizziness, depression, stomachache, backache, headache, and sleep disturbances, were all the most common among Hungarians. The monthly or more frequent prevalence of depression, stomach pain, headache, and sleep problems were significantly higher in Hungary than in the other Visegrad countries. Among the risk behaviors, we examined the prevalence of binge drinking and found that the highest prevalence of binge drinking episodes was among Hungarian youth. 

In Hungary, initiatives are being taken by health policy makers to reduce the prevalence of obesity and promote healthy lifestyles, but unfortunately these are not yet reflected in health indicators as expected. 

In Hungary, Act CXC of 2011 on National Public Education states that pupils must participate in at least one physical education lesson every school day [29]. To promote healthy eating, there is a school fruit and vegetable program and a school milk program, the implementation of which is regulated by the Ministry of Agriculture Decree 15/2021 (31.III.) and Decree 19/2021 (5.V.). The consumption of fruits, vegetables, milk, and milk products takes place several times a week in the institutions participating in the program [30,31]. In order to protect children’s health, school canteens have reduced the availability of soft drinks with added sugar or sweeteners, energy drinks, pre-packed products with added sugar or sweeteners, snacks with a high salt content, and snacks with a high saturated fat content [32]. Enhanced curricula and experiential learning can be effective strategies to promote healthy eating among adolescents [33].

Since 2012, the Hungarian Comprehensive School Health Promotion Program has been a requirement for all public education institutions in Hungary, focusing on healthy nutrition, daily physical education, health literacy, and mental health promotion, with the aim of ensuring that all children benefit from programs that effectively improve their health and promote their overall physical, mental, and emotional well-being. In schools, particular attention should be paid to various chemical and behavioral addictions [34].

To increase effectiveness, intervention programs should be adapted to the needs of adolescents with disabilities [19]. With regard to school support, special attention should be paid to the different needs of adolescents with high and low self-esteem [35]. Identifying and understanding barriers to healthier eating habits and adequate physical activity in this population is a key to the effectiveness of obesity prevention programs [36]. 

## 5. Conclusions

Hungary lagged behind the other Visegrad countries in most of the health behavior parameters examined. The prevalence of somatic complaints was significantly higher among Hungarian adolescents and mental well-being parameters were also worse. Children with disabilities experienced significantly higher rates of stress from schoolwork, which was associated with high rates of moodiness, and low rates of perceived good health. Pressure at school and frequent moodiness were phenomena that required urgent intervention to prevent later somatic complaints. The causes of the significantly higher rates of somatic complaints among young people in Hungary need to be identified in order to prevent later illnesses. The results suggest that further interventions are needed in Hungary and that differentiated, professional health promotion for young people with disabilities is required. Interventions based on interprofessional cooperation should be developed and implemented to protect mental health, taking into account the specific needs of young people with disabilities. It is clear that the coherence of health and youth policy objectives can make a significant contribution to the further development of health behavior. Community interventions based on interdisciplinary scientific evidence can only achieve their social goals if time-tested central regulations take into account the principle of subsidiarity. Accordingly, when defining cross-border health policy strategies in the Visegrad countries, indicators of the health behavior of Hungarian young people should also be taken into account in order to improve somatic and mental health. Specifically, complex planning and the organization of healthy diets, regular physical activity, and oral hygiene can reduce the risk of developing diseases.

### Limitations of Research

The lack of an established research methodology for research on children with disabilities made the research difficult to carry out. The presence of an interviewer was required in almost all cases during data collection, which may have biased the responses by preventing children with disabilities from expressing their true opinions in the presence of the interviewer. Last but not least, the small sample size does not allow for the generalization of results.

## Figures and Tables

**Table 1:** The main characteristics of sample of young Hungarians with disabilities N = 109.

Characteristics	%	
Gender	boy	57.8
	girl	42.2
Age	13 years	22.0
	15 years	41.3
	17 years	36.7
Intellectual disability (66 people)	mild	66.7
	intermediate	24.2
	serious	9.1
Mobility impairment (14 people)	mild	71.4
	intermediate	21.5
	serious	7.1
Visual impairment (15 people)	mild	73.3
	intermediate	20.0
	serious	6.7
Hearing impairment (7 people)	mild	71.4
	intermediate	28.6
	serious	0.0
Speech impairment (14 people)	mild	50.0
	intermediate	21.4
	serious	28.6
Autism spectrum disorder (23 people)	mild	74.0
	intermediate	8.6
	serious	17.4
Learning disorder (22 people)	mild	77.3
	intermediate	18.2
	serious	4.5
Type of settlement	city	66.1
	village	33.9
Child’s residence	at home with her family	80.7
	in college	9.2
	in a children’s home	2.8
	with a foster parent	7.3

(own editing).

**Table 2:** Life satisfaction among HUDC (%) N = 109.

0	1	2	3	4	5	6	7	8	9	10
0.9%	4.6%	1.8%	2.8%	2.8%	15.6%	11.0%	12.8%	15.6%	11.0%	21.1%

(own editing).

**Table 3:** Physical activity in the last 7 days among HUDC (%) N = 109.

0 Day	1 Day	2 Days	3 Days	4 Days	5 Days	6 Days	7 Days
22.9%	21.1%	11.0%	11.0%	4.6%	13.8%	5.5%	10.1%

(own editing).

**Table 4:** Frequency of vigorous physical activity among HUDC (%) N = 109.

Nothing	About Half an Hour	About 1 h	About 2–3 h	About 4–6 h	More Than 7 h
33.0	21.1	22.9	11.9	5.5	5.5

(own editing).

**Table 5:** Breakfast rates on school days among HUDC (%) N = 109.

Never	Once a Week	Twice a Week	Three Times a Week	Four Times a Week	Five Times a Week
14.7%	7.3%	11.0%	9.2%	3.7%	54.1%

(own editing).

**Table 6:** Frequency of different foods and drinks consumption among HUDC (%) N = 109.

Characteristics	Never	Less Often Than Weekly	Weekly	2–3 Times a Week	4–6 Times a Week	Daily	Several Times a Day	Total
Fruits	2.8%	19.3%	25.7%	21.1%	8.3%	16.5%	6.4%	100.1%
Vegetables	9.2%	12.8%	26.6%	18.3%	9.3%	16.5%	7.3%	100.0%
Sweets	4.6%	20.2%	14.7%	19.3%	11.0%	22.9%	7.3%	100.0%
Cola/Soft drinks	14.7%	13.8%	21.1%	17.4%	10.1%	12.8%	10.1%	100.0%

(own editing).

**Table 7:** Eating behaviors and oral health (%).

Characteristics	Hungary		Hungarian Disabled Children	
	N, NF, R%	95% CI	N, NF, R%	95% CI
Weekday breakfast on all five days	1697, 3739, 45.39%	[43.79–46.98]	59, 109, 54.13%	[44.7–63.48]
Sig.	proportion analysis NS			
Eat fruits more than once daily	670, 3758, 17.83%	[16.60–19.05]	7, 109, 6.42%	[1.82–11.02]
Sig.	proportion analysis NS			
Eat vegetables more than once daily	509, 3757, 13.55%	[12.45–14.64]	8, 109 7.34%	[2.44–12.24]
Sig.	proportion analysis NS			
Eat sweets more at least once a day	416, 3756, 11.08%	[10.07–12.08]	8, 109 7.34%	[2.44–12.24]
Sig.	proportion analysis NS			
Drinks Cola/soft drinks at least once a day	919, 3760 24.44%	[24.44–23.07]	25, 109 22.94%	[15.04–30.83]
Sig.	proportion analysis NS			
Tooth brushing more than once a day	2292, 3750 61.12%	[59.56–62.68]	31, 109 28.44%	[19.97–36.91]
Sig.	proportion analysis NS			

Sig.: Proportion Z-Tests for Pair of Countries; N = Number of Elements, NF = full, R = N/NF Ratio of Subgroup (%); CI = 95% Confidence Interval. (own editing).

**Table 8:** Physical activity, body image, health status (%).

Characteristic	Hungary		Hungarian Disabled Children	
	N, NF, R%	95% CI	N, NF, R%	95% CI
Physical activity every day for the last 7 days	727, 3694, 19.68%	[18.40–20.96]	11, 109, 10.09%	[4.44–15.75]
Sig.	proportion analysis NS			
Frequency of vigorous physical activity at least 4–6 times a week	1628, 3755, 43.36%	[41.77–44.94]	23, 109, 21.10%	[13.44–28.76]
Sig.	proportion analysis NS			
Overweight or obese	880, 3419, 25.74%	[24.27–27.20]	23, 109, 21.10%	[13.44–28.76]
Sig.	proportion analysis NS			
Thinness	420, 3417, 12.29%	[11.19–13.39]	7, 109, 6.42%	[1.82–11.02]
Sig.	proportion analysis NS			
Self-rated body image a bit too fat, much too fat	1199, 3674, 32.63%	[31.12–34.15]	25, 109, 22.94%	[15.04–30.83]
Sig.	proportion analysis NS			
Self-rated health status excellent	995, 3716, 26.78%	[25.35–28.20]	14, 109, 12.84%	[6.56–19.13]
Sig.	proportion analysis NS			

Sig.: proportion Z-tests for pair of countries; N = number of elements, NF = full, R = N/NF ratio of subgroup (%); CI = 95% confidence interval. (own editing).

**Table 9:** Family Context.

Characteristics	Hungary		Hungarian Disabled Children	
	N, NF, R%	95% CI	N, NF, R%	95% CI
Mother in main home	3526, 11,070, 94.08%	[93.32–94.83]	100, 109, 91.74%	[86.58–96.91]
Sig.	proportion analysis NS			
Father in main home	2781, 3748, 74.20%	[72.80–75.60]	76, 109, 69.72%	[61.10–78.35]
Sig.	proportion analysis NS			
Mother job	3233, 3747, 86.28%	[85.18–87.38]	63, 109, 57.80%	[48.53–67.07]
Sig.	proportion analysis NS			
Father job	3452, 3740, **92.30%**	[91.45–93.15]	80, 109, 73.39%	[65.10–81.69]
Sig.	χ^2^ test NS; proportion analysis HU-HUDC *p* < 0.001			

Sig.: Proportion Z-tests for pair of countries; N = number of elements, NF = full, R = N/NF ratio of subgroup (%); CI = 95% confidence interval. (own editing).

**Table 10:** Life satisfaction, School experience, Mental Well-Being (%).

Characteristics	Hungary		Hungarian Disabled Children	
	N, NF, R%	95% CI	N, NF, R%	95% CI
Life satisfaction best possible life	659, 3670, 17.96%	[16.71–19.20]	23, 109, 21.10%	[13.44–28.76]
Sig.	proportion analysis NS			
Feeling low at least once a month	2653, 3697, **71.76%**	[70.31–73.21]	66, 109, 60.55%	[51.38–69.73]
Sig.	χ^2^ test *p* < 0.001; proportion analysis HU-HUDC *p* = 0.011			
Stomachache at least once a month	2289, 3704, **61.80%**	[60.23–63.36]	49, 109, 44.95%	[35.62–54.29]
Sig.	χ^2^ test *p* < 0.0001; proportion analysis HU-HUDC *p* = 0.001			
Backache at least once a month	1684, 3702, 45.49%	[43.88–47.09]	33, 109, 30.28%	[21.65–38.90]
Sig.	proportion analysis NS			
Headache at least once a month	2437, 3708, **65.72%**	[64.20–67.25]	47, 109, 43.12%	[33.82–52.42]
Sig.	χ^2^ test *p* < 0.0001; proportion analysis HU-HUDC *p* < 0.001			
Feeling dizzy at least once a month	1323, 3690, 35.85	[34.31–37.40]	29, 109, 26.61	[18.31–34.90]
Sig.	proportion analysis NS			
Difficulties in sleeping at least once a month	2103, 3695, 56.91%	[55.32–58.51]	54, 109, 49.54%	[40.15–58.93]
Sig.	proportion analysis NS			
Pressured by schoolwork a lot	267, 3710, 7.20%	[6.37–8.03]	62, 109, **56.88%**	[47.58–66.18]
Sig.	χ^2^ test *p* < 0.0001; proportion analysis HUDC-HU *p* < 0.001			

Sig.: proportion Z-tests for pair of countries; N = number of elements, NF = full, R = N/NF ratio of subgroup (%); CI = 95% confidence interval. (own editing).

**Table 11:** Drunkenness (%).

Characteristics	Hungary		Hungarian Disabled Children	
	N, NF, R%	95% CI	N, NF, R%	95% CI
Drunk- ness lifetime 2–3 times	418, 3702, 11.29%	[10.27–12.31]	15, 109, 13.76%	[7.29–20.23]
Sig.	proportion analysis NS			

Sig.: proportion Z-tests for pair of countries; N = number of elements, NF = full, R = N/NF ratio of subgroup (%); CI = 95% confidence interval. (own editing).

**Table 12:** Eating behaviors and oral health (%).

Characteristics	Czech Republic		Hungary		Poland		Slovakia	
	N, NF, %	95% CI	N, NF, R%	95% CI	N, NF, R%	95% CI	N, NF, R%	95% CI
Weekday breakfast on all five days	6065, 11,470, 52.88%	[51.96–53.79]	1697, 3739, 45.39%	[43.79–46.98]	3173, 5181, **61.24%**	[59.9–62.57]	2125, 4621, 45.99%	[44.55– 47.42]
Sig.	χ^2^ test *p* < 0.001; proportion analysis PO-CZ *p* < 0.001; PO-HU *p* < 0.001; PO-SL *p* < 0.001							
Eat fruits more than once daily	2848, 11,482, **24.80%**	[24.01–25.59]	670, 3758, 17.83%	[16.60–19.05]	1139, 5199, 21.91%	[20.78–23.03]	1087, 4659, 23.33%	[22.12–24.55]
Sig.	χ^2^ test *p* < 0.0001; proportion analysis CZ-HU *p* < 0.001; CZ-PO *p* < 0.001							
Eat vegetables more than once daily	2008, 11,371, 17.66%	[16.96–18.36]	509, 3757, 13.55%	[12.45–14.64]	859, 5193, 16.54%	[15.53–17.55]	867, 4632, **18.72%**	[17.59–19.84]
Sig.	χ^2^ test *p* < 0.0001; proportion analysis SL-HU *p* < 0.001							
Eat sweets more at least once a day	961, 11,376, 8.45%	[7.94–8.96]	416, 3756, 11.08%	[10.07–12.08]	659, 5195, 12.69%	[11.78–13.59]	810, 4634, **17.48%**	[16.39–18.57]
Sig.	χ^2^ test *p* = 0.0007; proportion analysis SL-CZ *p* < 0.001; SL-HU *p* < 0.001; SL-PO *p* < 0.001							
Drinks Cola/soft drinks at least once a day	1505, 11,374, 13.76%	[13.76–13.13]	919, 3760, 24.44%	[24.44–23.07]	845, 5195, 16.27%	[15.26–17.27]	984, 4631, **21.25%**	[20.07–22.43]
Sig.	χ^2^ test *p* < 0.0001; proportion analysis HU-CZ *p* < 0.001; HU-PO *p* < 0.001; HU-SL *p* = 0.001							
Tooth brushing more than once a day	8476, 11,526, **73.54%**	[72.73–74.34]	2292, 3750, 61.12%	[59.56–62.68]	3594, 5204, 69.06%	[67.81–70.32]	2920, 4638, 62.96%	[61.57–64.35]
Sig.	χ^2^ test *p* < 0.001; proportion analysis CZ-H *p* < 0.001; CZ-PO *p* < 0.001; CZ-SL *p* < 0.001							

Sig.: χ^2^ test for all countries; proportion Z-tests for pair of countries; N = number of elements, NF = full, R = N/NF ratio of subgroup (%); CI = 95% confidence interval. (own editing).

**Table 13:** Physical activity, body image, health status (%).

Characteristics	Czech Republic		Hungary		Poland		Slovakia	
	N, NF, R%	95% CI	N, NF, R%	95% CI	N, NF, R%	95% CI	N, NF, R%	95% CI
Physical activity every day for the last 7 days	2109, 11,534, 18.29%	[17.58–18.99]	727, 3694, 19.68%	[18.40–20.96]	891, 5193, 17.16%	[16.13–18.18]	1074, 4660, 23.05%	[21.84–24.26]
Sig.	proportion analysis NS							
Frequency of vigorous physical activity at least 4–6 times a week	4572, 11,502, 39.75%	[38.86–40.64]	1628, 3755, 43.36%	[41.77–44.94]	1721, 5194, 33.13%	[31.85–34.41]	2362, 4607, **51.27%**	[49.83–52.71]
Sig.	χ^2^ test *p* < 0.0001; proportion analysis SL-CZ *p* < 0.001; SL-HU *p* < 0.001; SL-PO *p* < 0.001							
Overweight or obese	2369, 10,743, 22.05%	[21.27–22.84]	880, 3419, **25.74%**	[24.27–27.20]	1010, 4744, 21.29%	[20.13–22.45]	845, 3943, 21.43%	[20.15–22.71]
Sig.	χ^2^ test *p* = 0.0004; proportion analysis HU-CZ *p* < 0.001; HU-PO *p* < 0.001; HU-SL *p* < 0.001							
Thinness	1358, 10,747, 12.64%	[12.01–13.26]	420, 3417, 12.29%	[11.19–13.39]	594, 4744, 12.52%	[11.58–13.46]	516, 3943, 13.09%	[12.03–14.14]
Sign.	proportion analysis NS							
Self-rated body image a bit too fat, much too fat	2889, 11,394, 25.36%	[24.56–26.15]	1199, 3674, 32.63%	[31.12–34.15]	2036, 5208, **39.09%**	[37.77–40.42]	1159, 4744, 24.43%	[23.21–25.65]
Sig.	χ^2^ test *p* < 0.001; proportion analysis PO-CZ *p* < 0.001; PO-HU *p* < 0.001; PO-SL *p* < 0.001							
Self-rated health status excellent	278, 11,543, 24.08%	[23.30–24.86]	995, 3716, 26.78%	[25.35–28.20]	1145, 5204, 22.00%	[20.88–23.13]	1320, 4767, 27.69%	[26.42–28.96]
Sig.	proportion analysis NS							

Sig.: χ^2^ test for all countries; proportion Z-tests for pair of countries; N = number of elements, NF = full, R = N/NF ratio of subgroup (%); CI = 95% confidence interval. (own editing).

**Table 14:** Life satisfaction, school experience, mental well-being (%).

Characteristics	Czech Republic		Hungary		Poland		Slovakia	
	N, NF, R%	95% CI	N, NF, R%	95% CI	N, NF, R%	95% CI	N, NF, R%	95% CI
Life satisfaction best possible life	1819, 11,479, 15.85%	[15.18–16.51]	659, 3670, 17.96%	[16.71–19.20]	755, 5165, 14.62%	[13.65–15.58]	786, 4781, 16.44%	[15.39–17.49]
Sig.	proportion analysis NS							
Feeling low at least once a month	5812, 11,034, 52.67%	[51.74–53.61]	2653, 3697, **71.76%**	[70.31–73.21]	2743, 5153, 53.23%	[51.87–54.59]	1710, 4501, 37.99%	[36.57–39.41]
Sig.	χ^2^ test *p* < 0.001; proportion analysis HU-CZ *p* < 0.001; HU-PO *p* < 0.001; HU-SL *p* < 0.001							
Stomachache at least once a month	3989, 11,124, 35.86%	[34.97–36.75]	2289, 3704, **61.80%**	[60.23–63.36]	2681, 5176, 51.80%	[50.44–53.16]	2260, 4569, 49.46%	[48.01–50.91]
Sig.	χ^2^ test *p* < 0.0001; proportion analysis HU-CZ *p* < 0.001; HU-PO *p* < 0.001; HU-SL *p* < 0.001							
Backache at least once a month	5021, 11,112, 45.19%	[44.26–46.11]	1684, 3702, 45.49%	[43.88–47.09]	1801, 5118, 35.19%	[33.88–36.50]	1856, 4560, 40.70%	[39.28–42.13]
Sig.	proportion analysis NS							
Headache at least once a month	5894, 360, 52.47%	[51.55–53.39]	2437, 3708, **65.72%**	[64.20–67.25]	2594, 5181, 50.07%	[48.71–51.43]	2237, 4645, 48.16%	[46.72–49.60]
Sig.	χ^2^ test *p* < 0.0001; proportion analysis HU-CZ *p* < 0.001; HU-PO *p* < 0.001; HU-SL *p* < 0.001							
Feeling dizzy at least once a month	1870, 11,058, 16.91%	[16.21–17.61]	1323, 3690, 35.85%	[34.31–37.40]	1444, 5163, 27.97%	[26.74–29.19]	1509, 4532, 33.30%	[31.92–34.67]
Sig.	proportion analysis NS							
Difficulties in sleeping at least once a month	5563, 11,071, 50.25%	[49.32–51.18]	2103, 3695, **56.91%**	[55.32–58.51]	2433, 5165, 47.11%	[45.74–48.47]	2103, 4523, 46.50%	[45.04–47.95]
Sig.	χ^2^ test *p* < 0.0001; proportion analysis HU-CZ *p* < 0.001; HU-PO *p* < 0.001; HU-SL *p* < 0.001							
Pressured by schoolwork a lot	1249, 11,508, 10.85%	[10.29–11.42]	267, 3710, 7.20%	[6.37–8.03]	753, 5213, 14.44%	[13.49–15.40]	278, 4432, 6.27%	[5.56–6.99]
Sig.	proportion analysis NS							

Sig.: χ^2^ test for all countries; proportion Z-tests for pair of countries; N = number of elements, NF = full, R = N/NF ratio of subgroup (%); CI = 95% confidence interval. (own editing).

**Table 15:** Drunkenness (%).

Characteristics	Czech Republic		Hungary		Poland		Slovakia	
	N, NF, R%	95% CI	N, NF, R%	95% CI	N, NF, R%	95% CI	N, NF, R%	95% CI
Drunke- ness lifetime 2–3 times	1111, 11,464, 9.69%	[9.15–10.23]	418, 3702, 11.29%	[10.27–12.31]	424, 5195, 8.16%	[7.42–8.91]	375, 4515, 8.31%	[7.50–9.11]
Sig.	proportion analysis NS							

Sig.: χ^2^ test for all countries; Proportion Z-tests for pair of countries; N = number of elements, NF = full, R = N/NF ratio of subgroup (%); CI = 95% confidence interval. (own editing).

**Table 16:** Family context.

Characteristics	Czech Republic		Hungary		Poland		Slovakia	
	N, NF, R%	95% CI	N, NF, R%	95% CI	N, NF, R%	95% CI	N, NF, R%	95% CI
Mother in main home	10,464, 11,070, 94.53%	[94.10–94.95]	3526, 11,070, 94.08%	[93.32–94.83]	4972, 5164, 96.28%	[95.77–96.80]	3498, 3911, 89.44%	[88.48–90.40]
Sig.	proportion analysis NS							
Father main home	8173, 11,070, 73.83%	[73.01–74.65]	2781, 3748, 74.20%	[72.80–75.60]	4129, 5164, 79.96%	[78.87–81.05]	2888, 3911, 73.84%	[72.47–75.22]
Sig.	proportion analysis NS							
Mother job	10,164, 11,082, **91.72%**	[91.20–92.23]	3233, 3747, 86.28%	[85.18–87.38]	4132, 5189, 79.63%	[78.53–80.73]	3272, 3757, 87.09%	[86.02–88.16]
Sign.	χ^2^ test *p* < 0.001; proportion analysis CZ-HU *p* < 0.001; CZ-PO *p* < 0.001; CZ-SL *p* < 0.001							
Father job	10,193, 11,067, 92.10%	[91.60–92.61]	3452, 3740, 92.30%	[91.45–93.15]	4732, 5186, 91.25%	[90.48–92.01]	3514, 3758, 93.51%	[92.72–94.29]
Sig.	proportion analysis NS							

Sig.: χ^2^ test for all countries; Proportion Z-tests for pair of countries; N = number of elements, NF = full, R = N/NF ratio of subgroup (%); CI = 95% confidence interval. (own editing).

## Data Availability

The data presented in this study are available on request from the corresponding author (Agota Barabas). The data are not publicly available due to restrictions, e.g., privacy or ethical restrictions.
